# Dopaminergic organization of striatum is linked to cortical activity and brain expression of genes associated with psychiatric illness

**DOI:** 10.1126/sciadv.abg1512

**Published:** 2021-06-09

**Authors:** Robert A. McCutcheon, Kirsten Brown, Matthew M. Nour, Stephen M. Smith, Mattia Veronese, Fernando Zelaya, Martin Osugo, Sameer Jauhar, William Hallett, Mitul M. Mehta, Oliver D. Howes

**Affiliations:** 1 Department of Psychosis Studies, Institute of Psychiatry, Psychology, and Neuroscience, King’s College London, London, UK.; 2 Psychiatric Imaging Group, MRC London Institute of Medical Sciences, Hammersmith Hospital, Imperial College London, London, UK.; 3 Institute of Clinical Sciences, Faculty of Medicine, Imperial College London, London, UK.; 4 Max Planck UCL Centre for Computational Psychiatry and Ageing Research University College London, London, UK.; 5 Wellcome Centre for Human Neuroimaging, University College London, London, UK.; 6 Oxford University Centre for Functional MRI of the Brain (FMRIB), Oxford, UK.; 7 Centre for Neuroimaging Sciences, Institute of Psychiatry, Psychology, and Neuroscience, King’s College London, London, UK.; 8 Invicro Imaging Services, Burlington Danes Building, Du Cane Road, London, UK.

## Abstract

Dopamine signaling is constrained to discrete tracts yet has brain-wide effects on neural activity. The nature of this relationship between local dopamine signaling and brain-wide neuronal activity is not clearly defined and has relevance for neuropsychiatric illnesses where abnormalities of cortical activity and dopamine signaling coexist. Using simultaneous PET-MRI in healthy volunteers, we find strong evidence that patterns of striatal dopamine signaling and cortical blood flow (an index of local neural activity) contain shared information. This shared information links amphetamine-induced changes in gradients of striatal dopamine receptor availability to changes in brain-wide blood flow and is informed by spatial patterns of gene expression enriched for genes implicated in schizophrenia, bipolar disorder, and autism spectrum disorder. These results advance our knowledge of the relationship between cortical function and striatal dopamine, with relevance for understanding pathophysiology and treatment of diseases in which simultaneous aberrations of these systems exist.

## Teaser

Simultaneous PET-MRI illustrates the relationship between striatal dopamine and cortical activity.

## INTRODUCTION

The cognitive and behavioral effects of striatal dopamine signaling are related to its influence on large-scale neural activity. Recent work in rodents has begun to characterize the nature of the relationship between local striatal signaling and cortex-wide effects (*1*–*3*). Striatal dopamine release, secondary to optogenetic or chemogenetic activation of mesolimbic pathways (*2*, *3*) or hypothalamic self-stimulation (*1*), has been shown to affect cortical activity, as has selective overexpression of striatal D2 receptors (*4*). This correspondence between striatal dopamine and cortical activity is of relevance, given the frequent simultaneous disruption of striatal dopaminergic function and cortical function in neuropsychiatric illness (*5*, *6*) and also for understanding the broad consequences of dopamine modulating pharmacotherapies. The difficulties inherent in the simultaneous measurement of neurochemical signaling and neuronal activity mean that this “local to global” relationship has not been systematically explored in humans.

**Fig. 1:**
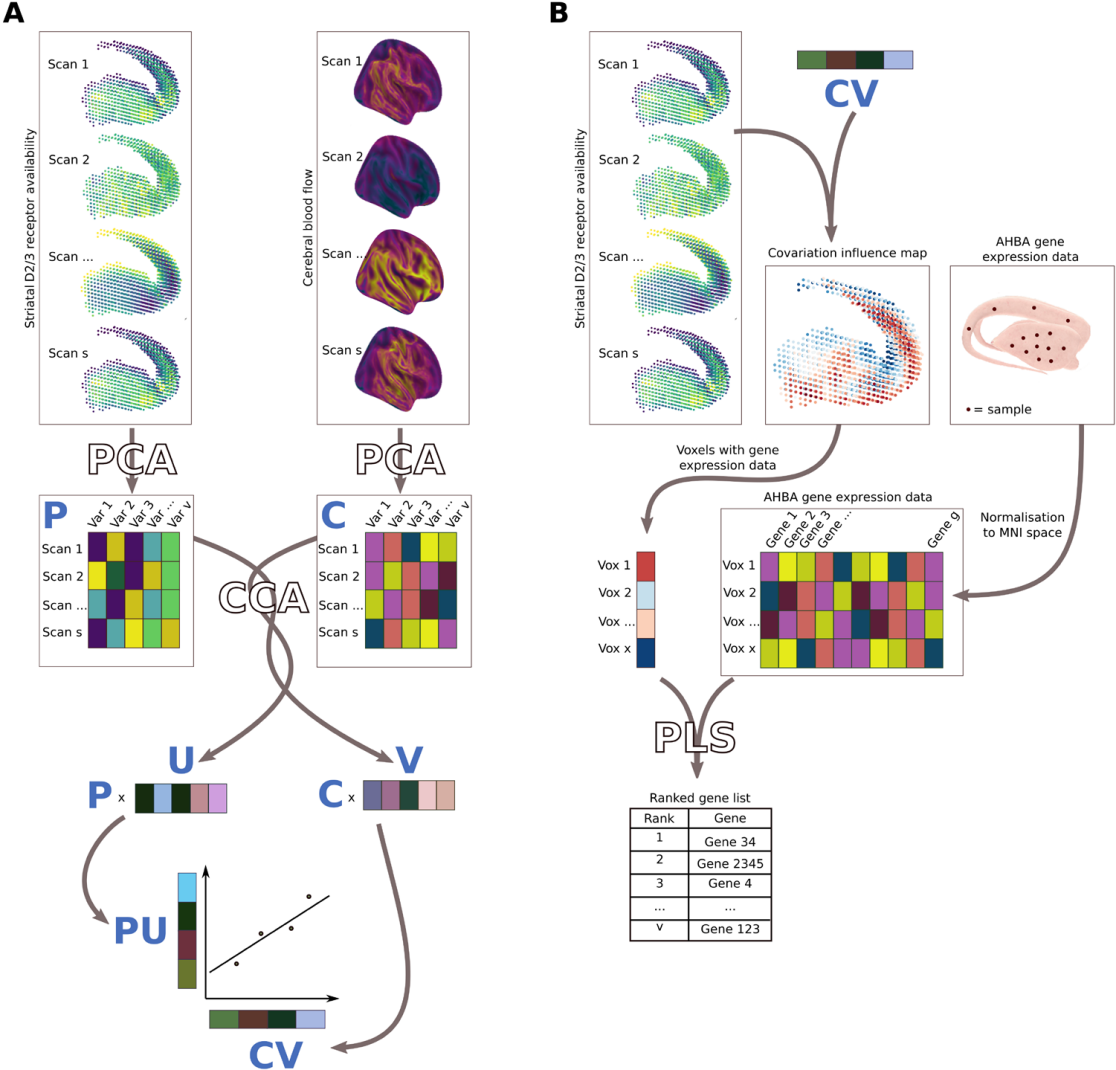
Overview of methods. (**A**) CCA is used to investigate the relationship between D2/3 receptor availability and CBF. The first step involves principal components analysis (PCA) to reduce the dimensionality of the data. CCA is then performed on these PCA matrices (number of rows = number of scans; number of columns = number of PCA-derived components). CCA calculates the canonical weight vectors **U** and **V** that maximize the correlation between canonical variates **PU** and **CV**. Each point in the scatter graph represents a single scan. (**B**) A striatal covariation influence map is calculated by correlating the canonical variate **PU** at each voxel of the maps of striatal D2/3 receptor availability. Gene expression data from the Allen Human Brain Atlas (AHBA) is normalized to Montreal Neurological Institute (MNI) space. Partial least squares (PLS) analysis is then used on voxels for which gene expression data exist to determine which genes show patterns of gene expression that most closely track the map of covariation influence.

In the current study, we use combined positron emission tomography and magnetic resonance imaging (PET-MRI) to simultaneously measure striatal D2/3 receptor availability using the radiotracer [11C]-(+)-PHNO, and cerebral blood flow (CBF) using arterial spin labeling (ASL) in healthy human volunteers (Fig. 1). [11C]-(+)- PHNO PET provides a reliable measure of striatal D2/3 receptors, with intraclass correlation coefficients of >0.9 (*7*, *8*). Furthermore, this measure is sensitive to physiologically and pharmacologically induced changes in synaptic dopamine concentration (*9*, *10*). CBF, as measured by ASL, covaries with acute changes in neural activity due to a tight neurovascular coupling in the cortex (*11*). Participants received two scans, one after administration of a placebo and the other after dexamphetamine; the latter intervention reliably induces increased synaptic dopamine levels via blockade of presynaptic dopamine transporters and increased firing rates of mesostriatal dopamine neurons (*12*, *13*).

We investigate the relationship between the two imaging modalities using canonical correlation analysis (CCA) to show that the spatial distribution of striatal dopamine receptor availability is linked to patterns of cortical activity. We also find that shared information identified using CCA links amphetamine-induced changes in gradients of striatal dopamine receptor availability to changes in brain-wide blood flow. Last, we show that this shared information between striatal dopamine and cortical activity is informed by patterns of striatal gene expression, and genes identified in this analysis are enriched for genes implicated in schizophrenia, bipolar disorder, and autism spectrum disorder.

## RESULTS

### Relationship between striatal D2/3 receptor availability and CBF

**Fig. 2:**
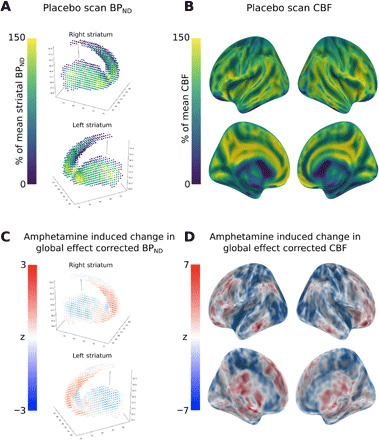
Striatal D2/D3 receptor availability and CBF measured with simultaneous PET-MRI. (**A**) Mean striatal BP_ND_ for placebo session scans. Before averaging across individuals, each individual participant scan was corrected for its mean value. (**B**) Mean CBF for placebo session scans. Before averaging across individuals, each individual participant scan was corrected for its mean value. (**C**) Change in mean value–corrected BP_ND_, *Z* scores represent results of a paired *t* test (amphetamine > placebo), and positive values (red) indicate greater relative BP_ND_ during the amphetamine scan. (**D**) Change in mean value–corrected CBF, *Z* scores represent results of a paired *t* test (amphetamine > placebo), and positive values (red) indicate greater relative CBF during the amphetamine scan.

Fifty-one simultaneous PET-MRI scans were obtained from 28 healthy volunteers. Twenty-three of these participants received two scans, with one following placebo administration and the other following administration of 0.5 mg of dexamphetamine per kilogram of dexamphetamine (randomized order, double-blind design). We mean-centered the PET and ASL values for each participant such that any emergent PET-ASL relationship was driven by the spatial distribution of D2/3 receptor availability and CBF (Fig. 2).

We sought to determine whether spatial patterns of D2/3 receptor availability across voxels of the bilateral striatum (PET voxel vectors) showed a relationship to patterns of CBF across voxels of the whole brain (ASL voxel vectors) between participants. We integrated the high-dimensional data from PET and MRI with the use of CCA, an analytic approach suited to uncovering many-to-many relationships in an unbiased manner (Fig. 1) (*14*). This extends previous attempts to uncover similar multimodal relationships, which typically reduce measures of dopamine function to a scalar value, thereby precluding characterization of the effects of dopaminergic spatial variation (*15*–*18*).

**Fig. 3:**
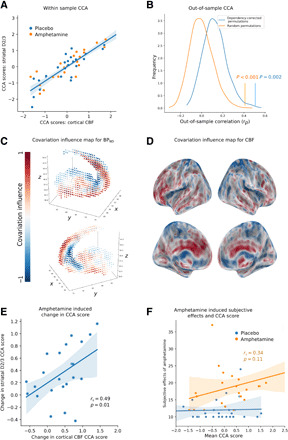
CCA identifies associations between striatal dopamine and cortical blood flow. (**A**) A scatterplot illustrating the mode of covariation between cortical blood flow and striatal D2/3 receptor availability, where each point represents an individual scan, and CCA has been performed using the entire sample. (**B**) The out-of-sample correlation predicted by the mode of covariation is statistically significant. The orange coloring represents results, where scan pairs were not allowed to split over train test partitions of the data, and the vertical line represents the observed correlation coefficient compared to a null distribution generated from 10,000 random subject-level permutations (*r*_p_ = 0.41, *P* < 0.001). The blue line represents the case in which permutations of the data used to calculate a null distribution maintained the linked dependency structure resulting from repeated scans (*r*_p_ = 0.51, *P* = 0.002). (**C**) Striatal voxels were colored according to their association, with the mode of covariation illustrated in (A), red indicates greater D2/3 receptor availability, and blue indicates lower D2/3 receptor availability, for high-scoring subjects (and vice versa for low-scoring subjects). (**D**) Cortical voxels were colored according to their association with the mode of covariation illustrated in (A); red indicates greater blood flow, and blue indicates lower blood flow, for high-scoring subjects (and vice versa for low-scoring subjects). An individual who had a pattern of cortical blood flow similar to (D) would be expected to show a pattern of striatal D2/3 receptor availability similar to (C). (**E**) The change in striatal D2/3 CCA scores following amphetamine administration is associated with a change in CBF CCA scores (*r*_s_ = 0.49, *P* = 0.01). (**F**) Average CCA scores (mean of striatal and cortical score) are not associated with the scores on the subjective effects of amphetamine scale in the sample as a whole (*r*_s_ = 0.26, *P* = 0.07) and when restricted to the amphetamine sessions (*r*_s_ = 0.34, *P* = 0.11).

**Fig. 4:**
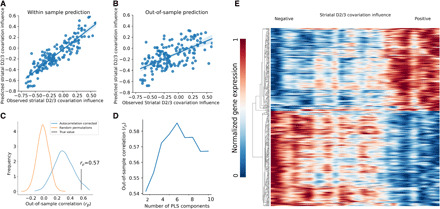
Gene expression predicts the influence of striatal tissue samples on the corticostriatal mode of covariation. (**A**) Partial least squares analysis identifies a pattern of gene expression associated with striatal D2/3 covariation influence (see Fig. 2C); each point represents a striatal voxel, with predicted striatal D2/3 covariation influence plotted against actual influence. (**B**) Average predicted out-of-sample striatal D2/3 covariation influence across 10 separate fivefold cross-validated partial least squares models compared to observed covariation influence; each point represents a striatal voxel. (**C**) The out-of-sample correlation between observed striatal D2/3 covariation influence and that predicted by partial least squares analysis of gene expression is represented by the vertical line. This is statistically significant compared to null models generated by 10,000 random permutations of striatal voxels (*P* < 0.001) and permutations where spatial autocorrelation is preserved (*P* = 0.044). (**D**) Out-of-sample prediction accuracy is greatest when using six partial least squares components, and this model is used in subsequent analyses. (**E**) Heatmap illustrating spatial patterns of gene expression for the 200 genes most strongly positively and negatively associated with striatal D2/3 covariation influence (*y* axis) against striatal voxels vectorized and arranged from left to right in order of covariation influence (*x* axis). Values smoothed with a 3-mm gaussian kernel in the *x* direction and clustered so as to visualize the positive-negative pattern of gene expression.

**Fig. 5:**
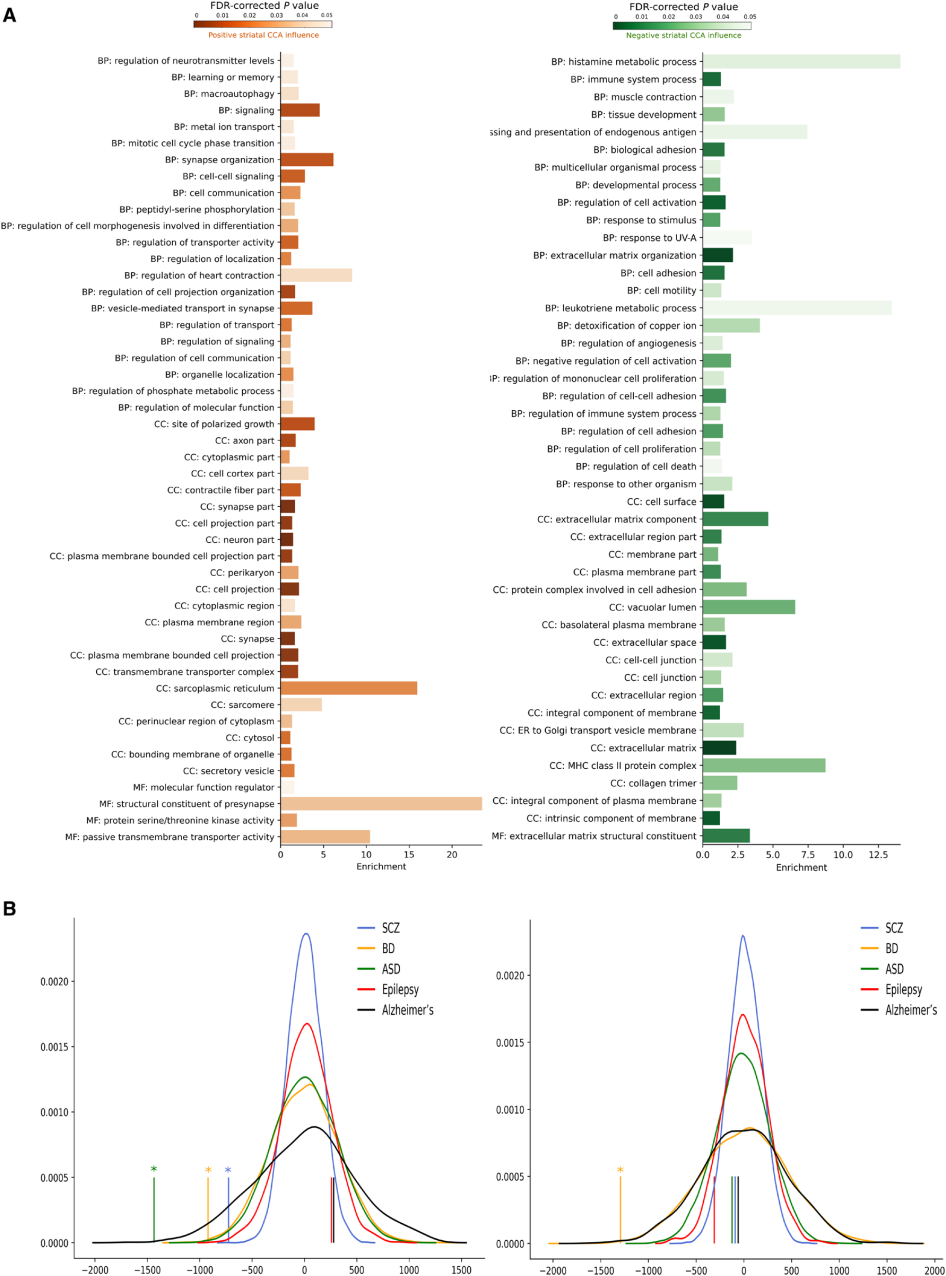
Genes associated with the corticostriatal mode of covariation are enriched for genes associated with synaptic proteins and with schizophrenia. (**A**) Enriched Gene Ontology terms associated with genes linked to the corticostriatal mode of covariation. Only terms with FDR-corrected significance at *P* < 0.05 and REViGO dispensability < 0.3 are shown. BP, biological process; CC, cellular component; MF, molecular function. (**B**) Figure on left shows that genes up-regulated in schizophrenia (*P* < 0.001), bipolar disorder (*P* = 0.004), and autism spectrum disorder (*P* < 0.001) are enriched among the genes associated with striatal D2/3 covariation influence. Figure on right shows that genes down-regulated in bipolar (*P* = 0.005) are enriched among the genes associated with striatal D2/3 covariation influence. Vertical lines indicate true median rank of disorder-related genes compared to null distributions representing 10,000 randomly selected gene sets. *P < 0.05.

CCA identifies two canonical weight vectors at the group level (one for each modality) that scale the influence of each voxel within the PET and ASL vectors, allowing a mapping for each subject from that participant’s original vectors to a pair of scalar values for that subject. We term these scalar values as “scores” for each participant, and across subjects, these scores constitute a pair of vectors, with each pair of entries representing the scores for a single participant. Each of these vectors is termed as a “canonical variate,” and we refer to a pair of canonical variates as a “mode of covariation.” CCA aims to identify weight vectors that maximize the correlation between these canonical variates.

We first sought to investigate whether there existed a mode of PET-ASL covariation that captured a significant multimodal relationship between subjects. Because CCA has the potential to identify relationships even in noise, we used a cross-validated analysis approach in which CCA was performed on a training sample containing 80% of the PET-ASL scan pairs, and the weights estimated here were then applied to as test sample containing the remaining scan pairs, with the correlation between canonical variates calculated only for this test sample. To test statistical significance, a null distribution was generated by following this same procedure for permuted samples in which the mapping between subjects’ ASL and PET images was shuffled, and the correlations were calculated for the permuted samples compared to that found for the original data.

Out-of-sample cross-validation and significance testing against 10,000 between subject permutations of the imaging data demonstrated a highly significant mode of covariation between the pattern of D2/3 receptor availability in the striatum and the pattern of CBF (mean out of sample *r*_p_ = 0.51, *P* < 0.001) (Fig. 3, A and B). This remained significant when ensuring that the dependence structure between placebo-amphetamine–linked scanning sessions was maintained within the permuted data (*P* = 0.002) (Fig. 3B) (*19*) and also when ensuring that placebo-amphetamine–linked scan pairs were not separated across train-test partitions of the data (*r*_p_ = 0.41, *P* < 0.001) (Fig. 3B). We also repeated this analysis restricting the CBF maps to a cortical mask to ensure that the covariation observed was not driven by blood flow changes within subcortical structures and found that the relationship remained highly significant (mean out of sample *r*_p_ = 0.44, *P* < 0.001). In a complementary analysis, this relationship between striatal dopamine and CBF was also demonstrated by the fact that, using a CCA model trained on leave-two-out data (i.e., two PET-ASL scan pairs left out), PET scans were able to predict ASL scans with 79% accuracy [compared to a mean null accuracy of 56% (SD of 9%) when CBF scans were permuted with dependency structure preserved; *P* = 0.001]. These findings consistently indicate that a mapping exists between patterns of striatal dopamine receptor availability indexed using [11C]-(+)-PHNO PET and patterns of cortical activity indexed using ASL-derived CBF maps.

### Spatial topography of the D2/3R-CBF relationship

We next sought to characterize the spatial profile of the identified mode of covariation. We characterized the strength of association between the identified mode of covariation and the CBF and D2/3 availability maps by correlating CBF and striatal D2/3 receptor availability at each voxel with their respective canonical variates (Fig. 3, C and D) (*20*). We term this correlation coefficient the “covariation influence,” and this value captures the degree to which each voxel influences the resulting canonical variate, thereby indicating the extent to which a voxel contributes to the observed striatal D2/3-CBF relationship. When considered at the individual participant level, participants with high cortical blood flow in an area of high cortical covariation influence will tend to show high D2/3 receptor availability in an area of high striatal covariation influence and low receptor availability in an area of low striatal influence (Fig. 3, C and D). This characterization of the spatial topography of the D2/3 receptor–CBF relationship provides a map of covariation influence that we then examine in subsequent analyses. The striatal map of covariation influence was distinct from the measure of D2/3 availability [placebo [11C]-(+)-PHNO nondisplaceable binding potential of the ligand (BP_ND_)] with a correlation of only *r*_p_ = 0.24, nor did it show evidence of an organization reflecting canonical resting-state connectivity patterns (*21*, *22*).

### Dopamine-CBF relationships predict changes in CBF following striatal dopamine release

We next explored the functional relevance of this relationship between striatal D2/3 receptor availability and CBF using an experimental perturbation of synaptic dopamine concentration. We used an amphetamine administration protocol, which reliably induces acute increases in synaptic dopamine concentration by increasing firing rates of ascending mesostriatal dopamine neurons and blocking presynaptic dopamine transporters (*12*, *13*). Striatal dopamine release can be quantified by the within-subject reduction in the nondisplaceable binding potential of competitive ligands such as [11C]-(+)-PHNO using PET. We examined whether the within-subject amphetamine-induced change in the striatal D2/3 CCA score was associated with the change in cortical CBF CCA score and found a statistically significant correlation (*r*_s_ = 0.49, *P* = 0.017) (Fig. 3E). This relationship was also present when using CCA scores calculated using a model that was fit solely using placebo data to avoid any potential circularity in the analysis (*r*_s_ = 0.78, *P* < 0.001) (fig. S3).

We examined whether participants’ mean CCA scores correlated with the scores on the subjective effects of amphetamine scale (*23*, *24*). No significant correlation between CCA score and subjective effects was present in either the sample as a whole (*r*_s_ = 0.26, *P* = 0.07) or when restricted to the amphetamine sessions (*r*_s_ = 0.34, *P* = 0.11) (Fig. 3F).

### Patterns of striatal gene expression shape the linkage between striatal dopamine receptor availability and CBF

We next investigated the genetic basis of the striatal D2/3 receptor–CBF relationship. To do this, we examined how spatial patterns of striatal gene expression relate to the pattern of striatal voxels showing a strong relationship with cortical blood flow (i.e., the covariation influence map; Fig. 3E). Normalized gene expression data for 15,633 genes were obtained from samples extracted from the striatum of six deceased human donors from the Allen Human Brain Atlas (AHBA). Each sample was matched to the voxel in which it lay and, if lying outside the striatal mask, was matched to the closest voxel as long as this was within 3 mm, resulting in 153 eligible samples. We used partial least squares regression to investigate the relationship between patterns of striatal gene expression and striatal covariation influence (Fig. 4) (*25*). Using out-of-sample cross-validation, we found that the pattern of striatal gene expression was predictive of the pattern of striatal covariation influence, and this was statistically significant when tested against 10,000 random permutations of covariation influence values (*r*_p_ = 0.57, *P* < 0.001) (Fig. 4, B and C) (*26*). We additionally used a more stringent test in which we preserved the spatial autocorrelation present in the map of covariation influence (*27*), and this also showed a statistically significant relationship between gene expression and covariation influence (*P* = 0.044) (Fig. 4C). We determined that a model using six partial least squares components gave the best out-of-sample prediction accuracy of covariation influence (Fig. 4D). These findings show that the D2/3-CBF relationship identified above is at least partially under genetic control.

### Genes associated with the linkage between striatal dopamine receptor availability and CBF are linked to cognition and psychiatric illness

We next sought to shed light on how the relationship between striatal dopamine and cortical activity may relate to neuropsychiatric diseases by examining the partial least squares model discussed above. We calculated the contribution of each gene to the six component partial least squares model using mean VIP [variable importance in the projection; (*25*)] scores from 10,000 bootstrapped runs (resampling with replacement of the 153 striatal samples). Enrichment analysis of this VIP ranked gene list demonstrated that genes most associated with positive covariation influence were enriched for genes implicated in a wide range of synaptic and neuronal processes and linked to cognition (Fig. 5A and tables S2 and S3) (*28*). When examining specific strongly associated genes, *KCNK1* and *KCNV1* showed the fifth and seventh strongest positive correlation between gene expression and covariation influence, respectively. These genes code for voltage-gated potassium channel subunits and have been linked to motivation deficits, schizophrenia, and autism spectrum disorder (*29*–*32*). Other strongly associated genes include *SYNPO* [which is involved in the regulation of synaptic plasticity, cognitive flexibility, and schizophrenia (*33*, *34*)] (table S2 for further details).

We next investigated whether genes associated with covariation influence were enriched for genes showing altered expression in schizophrenia, bipolar disorder, autism spectrum disorder, Alzheimer’s disease, and epilepsy. Akin to previous analyses (*35*, *36*), we examined whether the median ranking of disorder associated genes within the VIP ranked gene list was statistically different to the median ranking of 10,000 randomly selected gene sets. We found that genes negatively associated with covariation influence were enriched for genes up-regulated in schizophrenia (*P* < 0.001) (*37*), bipolar disorder (*P* = 0.004) (*37*), and autism spectrum disorder (*P* < 0.001) (*37*) (Fig. 5B); disorders that are all characterized by aberrant striatal dopamine signaling or corticostriatal connectivity (*5*, *38*, *39*). Genes up-regulated in epilepsy (*40*) and Alzheimer’s disease (*41*) did not show this association. When examining genes down-regulated in these disorders, it was only bipolar disorder that showed an association with genes associated with covariation influence (*P* = 0.005). Together, these results provide important convergent evidence that the identified relationship between D2/3R availability and CBF in our sample is of relevance to understanding the neurobiology of common and debilitating psychiatric disorders and generalizes to neurobiological findings beyond neuroimaging, collected in a separate cohort of individuals.

## DISCUSSION

In the current study, we use simultaneous PET-MRI to demonstrate a strong link between the spatial patterns of striatal dopamine D2/3 receptor availability and spatial patterns of CBF. Our findings are in keeping with recent preclinical findings that indicate that the neuromodulatory effects of striatal dopamine signaling extend well beyond sites of local release and suggest that the dopamine signaling in the striatum has effects in distal cortical regions (*1*–*3*).

After characterizing this relationship between striatal dopamine and CBF, we identified associations with amphetamine-induced effects and demonstrated patterns of striatal gene expression, which are associated with the striatal dopamine-CBF relationship. Furthermore, the genes identified showed enrichment for gene sets implicated in neuronal signaling, cognition, and several neuropsychiatric disorders. It is of interest that the identified genes were not enriched for Alzheimer’s disease or epilepsy. We are limited, however, in drawing inferences regarding the specificity of our findings to disorders with purportedly more neurodevelopmental origins, given the differences in methodology used in calculating gene expression levels between studies, and other potentially confounding factors such as medication exposure.

The relationship between dopamine and global cortical function has been studied extensively. This has often focused on the role of cortical dopamine receptors and the function these play in higher-order cognitive functions, although it has also been noted that mesostriatal dopamine signaling plays a role extending beyond movement gating and reward pathways (*17*, *42*, *43*). The bidirectional relationship between striatal dopamine signaling and global cortical function has been hypothesized on the basis of well-characterized corticobasal ganglia circuits (*42*), and recent work has provided further empirical support (*1*–*3*). Earlier attempts have often attempted to simplify investigation of this striatal dopamine-cortex relationship by condensing measures of dopamine or cortical function to a single dimension (*15*–*18*). The use of CCA, however, is less vulnerable to bias or to obscure the full nature of the relationship, as it is able to capture the many-to-many relations between striatum and cortex without artificially condensing either region to a single variable or making a priori judgments about which links should be prioritized. Simultaneous PET-MR reduces the variability that occurs if scans were conducted separately and allows for concurrent measurement of amphetamine effects on CBF and D2/3 receptor availability. In addition, the use of a pharmacological challenge demonstrates the functional relevance of this relationship, showing that changes in patterns of striatal dopamine receptor availability are associated with distal changes in patterns of CBF.

Further work of interest includes the use of preclinical models to investigate the causal influence of genes highlighted in the current study, specifically as to potential manipulation of striatum-cortex relationships. In addition, work in humans should include whether the striatum-cortex relationships identified in the current study predicts the cortical effects of pharmacological compounds that show spatially selective modulation of striatal dopamine signaling (*5*). When combined with work in patient populations mapping the relationship between striatal dopamine and cortical function, this has the potential to aid the development of therapeutics targeted at correcting a broad spectrum of neurobiological abnormalities.

CBF is a marker of neuronal activity that has reduced temporal resolution compared to the more commonly used blood oxygen level-dependent (BOLD) signal. CBF is, however, preferable for this study as the signal originates from capillaries without the contribution of draining veins, which results in greater spatial specificity compared to BOLD (*44*). Although BOLD may be preferable for the investigation of task-based activity connectivity, ASL is ideally suited to obtaining a measure of tonic neural activity. As with BOLD, although strongly coupled to neural activity, CBF is a proxy measure, and hence, there exists the possibility for confounds to influence the observed findings. However, the fact that we controlled for global changes to both receptor availability and blood flow indicate that our findings are independent of global cerebral blood circulation. It is also a possibility that factors other than underlying neurobiology, e.g., image preprocessing steps, may influence downstream results.

The current findings raise the possibility that aberrant cortical functioning observed in several neuropsychiatric disorders may, in some cases, reflect abnormalities in striatal dopamine signaling. This interpretation is supported by our finding that the striatal regions most strongly associated with the mode of covariation were enriched for genes overexpressed in several psychiatric disorders. Furthermore, it suggests that pharmacological interventions that are able to modulate striatal dopamine signaling in a regionally specific manner may also have the potential to selectively modulate cortical activity, and further research may investigate the therapeutic possibilities available here.

## MATERIALS AND METHODS

### Experimental design

Fifty-one simultaneous PET-MRI scans were obtained from 28 participants. Twenty-three of these participants received two scans, with one following placebo administration and the other following administration of dexamphetamine (0.5 mg/kg). The relationship between PET measures of striatal dopamine receptor availability and ASL measures of CBF was analyzed using CCA. We then investigated the relationship between the CCA measures and the effects of amphetamine. The CCA analysis produces a map of striatal covariation influence, and we investigated whether this map was informed by underlying patterns of striatal gene expression. We characterized genes implicated by this analysis using Gene Ontology (GO) enrichment analysis and also investigated whether an association existed with genes implicated in several psychiatric disorders.

### Participants

Participants were recruited via online advertising. All participants gave informed written consent. Inclusion criteria were age above 18 years and capacity to give written informed consent. Exclusion criteria were (i) any past or current major medical condition, (ii) history of a neurological or psychiatric disorder (including substance abuse/dependence) as determined by the Structured Clinical Interview for Diagnostic and Statistical Manual of Mental Disorders (DSM-IV) and medical review, (iii) history of head injury with a loss of consciousness, (iv) a family history of any psychiatric disorder in first-degree relatives, and (v) contraindications to PET or MRI scanning (significant prior exposure to radiation, pregnancy, or breast feeding) or amphetamine.

Participants attended for a scan on two separate occasions, separated by a minimum of 3 days. On one occasion, participants received an oral placebo (101 mg of lactose/sucrose tablets; number of tablets matched to number administered when receiving dexamphetamine) before scanning, and on the other, they received a dose (0.5 mg/kg) of oral dexamphetamine. The order of drug/placebo scans was randomized. Dosing occurred 3 hours before scanning so that in the case of dexamphetamine, peak drug levels coincided with scan time (*45*). The order of scans (i.e., dexamphetamine/placebo order) was randomized, and participants and staff involved in assessment were blinded to this order. The subjective effects of amphetamine scale (*23*, *24*) was given to participants at baseline for 1.5 and 3 hours to assess the psychological effects of amphetamine. The scale involves ranking each of 10 possible drug effects on a five-point scale ranging from “least” to “most.” Positive effects include “high,” “rush,” “good effects,” “liking,” and “desire for drug”; negative effects included “fidgety,” “anxious,” “dizziness,” “dry mouth,” and “distrust.” Measures obtained at the 1.5-hour time point were used in subsequent analysis, as this was the time point with the most data obtained and at which subjective effects were greatest. The study was approved by the local National Health Service (NHS) Research Ethics Committee (12/LO/1955) and the Administration of Radioactive Substances Advisory Committee.

### Image acquisition

Neuroimaging data were acquired using a GE SIGNA simultaneous PET-MRI scanner. [11C]-(+)-PHNO (0.020 to 0.029 μg/kg) was injected immediately before the commencement of scanning as a smooth bolus injection over 30 s. During the initial minutes of data acquisition, the PET signal changes rapidly, and PET modeling will be particularly sensitive to the integrity of the data. The heat generated by the gradient coils during MRI acquisition was noted to have a small effect upon the PET signal, so a 10-min MRI-free period at scan start was used. This initial 10-min period of PET-only data collection was followed by simultaneous PET-MRI acquisition. Mean injected activity was 140 megabecquerel.

#### Structural MRI

A three-dimensional (3D) BRAVO T1-weighted (T1w) structural scan was obtained with the following parameters: flip angle = 12°, inversion time (TI) = 400 ms, echo time (TE) = 3.2 ms, repetition time (TR) = 8.5 ms, matrix = 256 × 256, number of slices = 188, and 1-mm isotropic voxels.

#### Arterial spin labeling

Images were acquired using a 3D pseudo-continuous ASL sequence, and 38 slices of a 128 × 128 matrix were obtained, resulting in a spatial resolution of 1.88 mm by 1.88 mm by 4 mm. Parameters were as follows: TE/TR = 10.7/4854 ms, labeling duration = 1450 ms, postlabeling delay = 2025 ms, interslice gap = 0, bandwidth = 62.5 kHz, flip angle = 111°, and number of acquisitions = 4. A proton density image was acquired using the same parameters to compute the CBF map in standard physiological units (milliliters of blood per 100 g of tissue/min).

Automatic CBF map reconstruction used the following quantification algorithm

CBF=6000*λ(1−exp(−ST(s)T1I(s)))exp(PLD (s)T1b(s))2T1b(s)(1−exp(−LT(s)T1b(s)))ε*NEXPW(PWSF PPWD)

*T*_1*b*_ is the *T*_1_ of blood (1.6 s at 3 T). The partial saturation of the proton density image (PD) is corrected using a typical gray matter value for *T*_1*t*_ of 1.2 s. The partition coefficient λ is set to the whole-brain average of 0.9. Efficiency, ε, is set to 0.6. PLD refers to the postlabeling delay, and labelling time (LT) refers to the labeling duration. PW is the perfusion-weighted image. SF_PW_ is the scaling factor of the sequence, and NEX_PW_ is the number of excitations. As only a final label-control difference image is produced by the scanner, motion correction cannot be applied retrospectively. However, this should not markedly affect the data quality because background suppression and the alternation between control and labeled phases reduce motion artifact [see (*46*) for full details].

#### Positron emission tomography

Dynamic emission data were acquired for 90 min following radiotracer administration. A zero echo time (ZTE)–based MR attenuation correction map with the following parameters was obtained: flip angle = 0.8°, matrix = 110 × 110 × 116, voxel size = 2.4-mm isotropic, number of averages = 4, bandwidth = ±62.5 kHz, and acquisition time = 42 s.

Dynamic PET images were reconstructed on the PET/MR scanner using VPFX-S, a fully 3D ordered subset expectation maximization algorithm with time-of-flight information and resolution recovery, six iterations, 16 subsets, no postreconstruction smoothing, matrix of 128 × 128 × 89, and voxel size of 2 mm by 2 mm by 2.78 mm, with corrections applied for detector normalization, randoms, scatter, dead time, and radioactive decay. Attenuation correction was performed using the ZTE map implemented using GE scanner software (*47*).

### Image processing

#### Structural MRI

Normalization of the structural T1w to MNI space was implemented using fMRIPrep 20.0.5 (*48*), which is based on Nipype 1.4.2 (*49*). All available T1w images were corrected for intensity nonuniformity (INU) with N4BiasFieldCorrection (*50*) distributed with Advanced Normalization Tools (ANTs) 2.2.0 (*51*). The T1w reference was then skull-stripped with a Nipype implementation of the antsBrainExtraction.sh workflow (from ANTs) using OASIS30ANTs as target template. Brain tissue segmentation of cerebrospinal fluid, white matter, and gray matter was performed on the brain-extracted T1w using fast (*52*). A T1w reference map was computed after registration of T1w images (after INU correction) using mri_robust_template (*53*). Brain surfaces were reconstructed using recon-all [FreeSurfer 6.0.1; (*54*)], and the brain mask estimated previously was refined with a custom variation of the method to reconcile ANT-derived and FreeSurfer-derived segmentations of the cortical gray matter of Mindboggle (*55*). Volume-based spatial normalization to MNI standard spaces [MNI152NLin2009cAsym; (*56*)] was performed through nonlinear registration with antsRegistration (ANTs 2.2.0) using brain-extracted versions of both T1w reference and the T1w template.

#### Arterial spin labeling

Proton density maps were linearly coregistered to structural T1w images, and this transform subsequently applied to the CBF maps. A nonlinear transform from T1w native space to MNI space (derived from the normalization of the structural T1w described above), was then applied to the CBF maps. Global CBF was calculated by calculating the mean CBF for voxels within a brain mask, and relative maps were then derived by dividing all voxels by this figure to account for peripheral (global) drug effects and between-subject variability in global perfusion. Subsequent analyses used these unsmoothed relative maps after masking with a group mask computed using the nilearn function “compute_multi_gray_matter_mask” (*57*). This resulted in 51 images each containing 229,007 voxels.

#### Positron emission tomography

PET images were analyzed using the Molecular Imaging And Kinetic Analysis Toolbox (version 4.3.13). A frame-by-frame registration process on a single frame of reference was used for motion correction. Time activity curves for each voxel were generated, and the simplified reference tissue model with cerebellum gray matter as a reference region was applied to these curves to estimate the nondisplaceable binding potential of the ligand (BP_ND_) (*58*, *59*).

Unsmoothed voxel-wise BP_ND_ maps in native space were then coregistered to the structural T1w images, and the nonlinear transform from T1w native space to MNI space (described above) was applied to these BP_ND_ maps. A previously defined mask was used to restrict the images to striatal voxels (*60*), and as with CBF, each voxel within a participant BP_ND_ map was divided by that participant’s mean striatal BP_ND_ to account for global effects. This resulted in 51 images, each containing 2847 voxels.

### Canonical correlation analysis

CCA is a technique ideally suited to the simultaneous evaluation of two highly multidimensional sets of variables. CCA allows the identification of many-to-many relations in contrast to techniques such as multiple linear regression that requires one set of variables to be condensed to a single variable. For an intuitive understanding, CCA can be understood as an extension of principal components analysis (PCA). PCA decomposes a single set of variables to a smaller number of components that capture the variables’ latent sources of variation, while CCA seeks to simultaneously decompose two sets of variables to maximize the correlation between these two sets (see Fig. 1) (*14*). For a full description of the technique, with an emphasis on applications in neuroimaging, please see the review by Wang *et al.* (*14*).

For both CBF data (229,007 voxels × *N*) and PET data (2847 voxels × *N*), where there are data from *N* = 51 scans, dimensionality reduction via PCA was undertaken before running CCA (see figs. S1 and S2) so as to avoid the overfitting that occurs if the number of features is significantly greater than the number of subjects. As PCA is affected by the relative scaling of each variable, standardization of each variable was performed by subtracting its mean and dividing by its SD. The optimal number of components is unclear, and PCA was therefore performed to give between *K* ∈ [2,3,4, …,10] components for both CBF and PET measures. This results in nine pairs of [*N*, *K*] matrices **C** and **P**, where **C** contains features derived from the CBF data and **P** contains features derived from the PET data. For each pair, the number of rows is equal to the number of scans (*N* = 51), while the number of columns is equal to the number of PCA components. For the PET data, the first 10 components explained 61% of the variance, while for CBF data, 42% were explained.

CCA was implemented using the python library “scikit-learn” (*61*). CCA calculates a pair of [1, *K*] canonical weight vectors **U** and **V** (which are each the length of the number of components), which maximize the correlation between **PU** and **CV**. **PU** and **CV** are vectors termed canonical variates, and each will be the length of the total number of scans. A pair of canonical variates is termed a mode of covariation. Given that CCA intentionally maximizes the correlation between canonical variates, statistical significance cannot be determined solely on the basis of the magnitude of a correlation coefficient between these canonical variates.

An out-of-sample cross-validation testing approach was used to test the statistical significance of the mode of covariation identified between striatal dopamine receptor availability and CBF. For each choice of PCA dimensionality, *K*, the data were randomly split 100 times into sets where 80% of the data were used for training and 20% for testing. For each train-test split, we conducted PCA with *K* components followed by CCA on the training data. The PCA and CCA models that were fit on the training data were then applied to the test data, and the correlation between the resulting test data canonical variates for the first CCA component was calculated. We used the mean correlation coefficient over 100 train-test splits as an estimate of the true out-of-sample performance for that PCA dimensionality. An overall correlation coefficient was then calculated by taking the mean correlation coefficient for the nine (*K* ∈[2,3,4, …,10]) dimensionalities. We used this method to avoid potential biases that could result from manually setting the number of PCA dimensions. The statistical significance of this correlation coefficient was calculated by comparing it to a null distribution generated by following the same procedure 10,000 times but after permuting CBF images to shuffle the mapping between CBF images and participant identity (i.e., instantiating the null that there is no shared information between striatal D2/3R availability and cortical CBF within subjects).

For most of the participants, there exist two scans (one following placebo, and the other following amphetamine). There, therefore, exists the potential for information leakage between training and test sets, which could erroneously inflate the statistical significance of results. We used two strategies to address this. First, we used a well-established approach in which the same dependency structure was kept when generating a null distribution (*19*). As a result, while the observed out-of-sample correlation coefficient may be inflated secondary to dependency structure, an equal inflation will also apply to the null distribution and therefore the *P* value will be unaffected. Second, in a complementary analysis, we restricted the shuffling of participants when partitioning the data into train and test sets so that placebo and amphetamine scans for a single subject remain together for each subject and are not separated across test and train sets, meaning that no dependency exists between test and train sets. To ensure that the observed relationship reflected cortex-wide relationships and was not driven by subcortical changes in blood flow, we also performed the above analysis while restricting the analysis to CBF data from cortical regions defined using the Harvard-Oxford cortical atlas (*62*).

In a complementary analysis, CCA (and PCA) was performed as on a training sample that randomly left out two pairs of PET-ASL scans. This model was then used to perform CCA on the two pairs of left out test scans. If the CCA scores for CBF and PET were ranked identically, e.g., the first pair of scans having higher scores for both CBF and PET, then this indicates that the CCA has been able to correctly match the pairs of scans and that this was counted as a successful match. If the ranking differed between modalities, then this indicated an unsuccessful match. Accuracy was quantified as the percentage of successful matches out of 100 samples. This was compared to null distributions generated as above by both randomly selecting subjects and selections in which the pair structure was maintained.

After establishing a statistically significant relationship between cortical activity and striatal dopamine, we then sought to investigate the nature of this relationship in greater depth. To do this, we first set the number of PCA components to eight as that had gave the most predictive out of sample relationship between the PET and CBF measure. We then fit CCA on the entire sample (resulting full loading matrices in table S1) and calculated the contribution of each voxel to by correlating CBF and PET measures with their respective canonical variate at each voxel (i.e., CBF voxels to the CBF canonical variate and PET voxels to the PET canonical variate). This is illustrated by the maps of covariation influence in Fig. 3 (C and D), where the intensity of shading represents the magnitude of correlation coefficient.

### Overlap of covariation influence maps with canonical functional connectivity networks

We investigated the overlap between known functional connectivity networks and the map of covariation influence. For the striatum, we investigated the overlap with the seven network parcellation described by Choi *et al.* (*21*), while for the cortex we used the seven network parcellation described by Yeo *et al.* (*22*). For each network, we calculated the observed overlap by averaging the value of covariation influence map voxels that were within that network. We then compared this observed value to a null distribution generated from 1000 null covariation influence maps. In the case of the striatum, these were generated using the BrainSMASH (brain surrogate maps with autocorrelated spatial heterogeneity) python toolbox so as to account for patterns of spatial autocorrelation (*27*). In the case of the cortex, the same approach was too computationally intensive to be undertaken so the null covariation influence maps were generated by permuting CBF and PET maps before running the CCA.

### Linking CCA measures and the effects of amphetamine

We next examined whether the amphetamine-induced change in the striatal D2/3 CCA score was associated with the change in cortical CBF CCA score, and we also performed a complementary analysis in which the PCA and CCA models were fit solely on placebo scans so as to avoid any potential circularity in the analysis. We also examined whether mean CCA scores were associated with participant scores on the subjective effects of amphetamine scale (*23*, *24*). In both cases, we used spearman’s correlation coefficient so that outliers would not unduly influence any potential association.

### Gene expression data

Gene expression data were obtained from six post mortem adult brains from the AHBA transcriptomic dataset (http://human.brain-map.org; RRID: SCR_007416) (*63*). Since the AHBA only includes data relating to two subjects for the right hemisphere, right-sided striatal samples were limited, and in keeping with similar analyses, we only consider left hemisphere samples (*36*).

The AHBA microarray expression data need to be both linked to regions of interest and combined across donors. To ensure robust and replicable results, we used the “abagen” (version 0.0.5) python toolbox, an externally developed and validated pipeline, which undertakes the steps described below (*64*).

The original MNI coordinates provided with the AHBA do not account for nonlinear deformations in their transformation from native to MNI space. We therefore used “corrected” MNI coordinates to match tissue samples to MNI space; this involves using ANTs to perform a more accurate nonlinear coregistration of each donor’s scan to an MNI template as opposed to the AHBA default based on an affine coregistration of a single donor brain.

Probe-to-gene mappings were reannotated with information from Arnatkevic̆iūtė *et al.* (*64*). This was followed by intensity-based filtering to remove probes that did not exceed background noise in 50% of all tissue samples. A representative probe was selected for multiple probes indexing the same gene based on which probe had the highest differential stability among donors. Each sample was matched to the voxel in which it lay and, if lying outside the striatal mask, was matched to the closest voxel as long as this was within 3 mm. This resulted in 153 eligible samples. Before aggregation across donors, expression values were normalized for each sample across genes for each donor using a scaled robust sigmoid normalization function.

### Genes associated with covariation influence

We used partial least squares regression to investigate associations between the pattern of striatal covariation influence illustrated in Fig. 3C and the pattern of striatal gene expression derived from the AHBA. Partial least squares regression is closely related to CCA in that it seeks to identify latent variables (the partial least squares components) that maximize correlation between collinear predictor variables (gene expression values in this case) and response variables (the covariation influence maps). Partial least squares incorporates dimensionality reduction directly and is suitable for predicting a univariate outcome variable. For model fitting, we used the Nonlinear Iterative Partial Least Squares “NIPALS” algorithm implemented in the python package scikit-learn. Out-of-sample prediction accuracy was determined by calculating the Pearson correlation between covariation influence predicted on the basis of gene expression and true covariation influence. For each component, accuracy was averaged across five sets of fivefold cross-validation. As with the use of CCA above, there is effectively a hyperparameter in that flexibility exists when choosing the number of components for dimensionality reduction. As above, to avoid bias, we fit models using 2 to 10 components and calculated the mean score of results.

Statistical significance of out-of-sample prediction accuracy was first tested by comparing these results to those obtained after randomly permuting the covariation influence values 10,000 times. This approach to significance testing is often used in studies linking gene expression data to neuroimaging measures (*26*, *65*) but does not account for the typically high degree of spatial autocorrelation observed in the brain maps under investigation (*27*). We therefore used the BrainSMASH python toolbox to generate 10,000 surrogate maps of covariation influence in which spatial autocorrelation was preserved and compared the accuracy of the gene expression data in predicting these surrogate maps to the original true map.

### Gene enrichment analysis

The contribution of each gene to the striatal covariation influence was calculated on the basis of that gene’s VIP score. The error in estimating each gene’s VIP score was assessed by bootstrapping (resampling with replacement of the 153 striatal voxels), *z* scores were then calculated as the ratio of each gene’s VIP to its bootstrap standard error, and this was used to rank the genes according to their level of influence on the striatal covariation influence map.

GO enrichment analysis was used to characterize the nature of the genes associated with the striatal covariation influence. To avoid artefactual elevation of GO terms for brain expression, we used previously reported methods (*35*) to first subset the VIP ranked gene list to include only genes showing above zero expression in cerebral cortex, amygdala, basal ganglia, cerebellum, cerebral cortex, hippocampal formation, and hypothalamus as listed within Human Protein Atlas RNA GTEx brain region gene expression database (www.proteinatlas.org/download/rna_brain_gtex.tsv.zip; table S3). Enrichment analysis was then performed for this brain refined, and VIP score ordered list was inputted to GOrilla (separately in increasing and decreasing order) (*28*). Resulting gene categories were considered significant if meeting a threshold of false discovery rate (FDR)–corrected *P* < 0.05 and showed a minimum of three overlapping genes. REViGO (http://revigo.irb.hr) was then used to cluster categories using the SimREl algorithm (*66*).

Using previously reported methods (*35*, *36*), we next explored whether the VIP ranked gene list was enriched for genes associated with neuropsychiatric illnesses. We used the findings of the psychENCODE consortium to identify genes that are up-regulated or down-regulated in autism spectrum disorder, schizophrenia, and bipolar disorder (*37*) and also genes differentially expressed in temporal lobe epilepsy (*40*) and Alzheimer’s disease (*41*). We first calculated the median rank of the disease-associated gene set within the VIP gene list and took the absolute value of how far this lay from the center of the list. We then compared this value to 10,000 randomly selected gene sets of the same size.

### Statistical analysis

Permutation testing of cross-validated out-of-sample association measures was the primary means of significance testing. These methods are described in detail above.

## SUPPLEMENTARY MATERIALS

Supplementary material for this article is available at http://advances.sciencemag.org/cgi/content/full/7/24/eabg1512/DC1

## Appendix

View/request a protocol for this paper from *Bio-protocol*.
